# Molecular Imaging of Experimental Abdominal Aortic Aneurysms

**DOI:** 10.1155/2013/973150

**Published:** 2013-04-23

**Authors:** Aneesh K. Ramaswamy, Mark Hamilton, Rucha V. Joshi, Benjamin P. Kline, Rui Li, Pu Wang, Craig J. Goergen

**Affiliations:** Weldon School of Biomedical Engineering, Purdue University, West Lafayette, IN 47907, USA

## Abstract

Current laboratory research in the field of abdominal aortic aneurysm (AAA) disease often utilizes small animal experimental models induced by genetic manipulation or chemical application. This has led to the use and development of multiple high-resolution molecular imaging modalities capable of tracking disease progression, quantifying the role of inflammation, and evaluating the effects of potential therapeutics. *In vivo* imaging reduces the number of research animals used, provides molecular and cellular information, and allows for longitudinal studies, a necessity when tracking vessel expansion in a single animal. This review outlines developments of both established and emerging molecular imaging techniques used to study AAA disease. Beyond the typical modalities used for anatomical imaging, which include ultrasound (US) and computed tomography (CT), previous molecular imaging efforts have used magnetic resonance (MR), near-infrared fluorescence (NIRF), bioluminescence, single-photon emission computed tomography (SPECT), and positron emission tomography (PET). Mouse and rat AAA models will hopefully provide insight into potential disease mechanisms, and the development of advanced molecular imaging techniques, if clinically useful, may have translational potential. These efforts could help improve the management of aneurysms and better evaluate the therapeutic potential of new treatments for human AAA disease.

## 1. Introduction

Abdominal aortic aneurysm (AAA) is an expansion and weakening of the abdominal aortic wall [1], typically defined as a pathological dilation of at least 50% [2]. It is estimated that AAAs affect somewhere between 0.5% and 3.2% of the US population [3], but incidence rates rise to between 4% and 7% in men over the age of 65 [4]. Aortic aneurysms that are typically detected through screening are small, requiring only periodic observations [5]. However, once the vessel diameter grows beyond 5.5 cm, the risk of vessel rupture increases drastically. Rupture of an AAA is often a lethal event and, in many cases, the first and only clinical symptom of pathological aortic expansion [6]. Death caused by AAA rupture is estimated at about 14,000 per year, though this figure may be an underestimation [7]. While open surgical intervention is currently the standard treatment for high-risk AAA, less invasive endovascular aneurysm repair (EVAR) is developing into a second common treatment procedure [8].

Small animal models of AAA have been established to characterize mechanistic properties of human AAA progression [9]. These small animal models closely mimic pathophysiology of human AAA, including increased matrix metalloproteinase (MMP) activity, inflammation, extracellular matrix (ECM) degradation, smooth muscle cell apoptosis, and neovascularization [10]. Currently, murine models are most frequently used because of cost considerations, well-documented genetic backgrounds, and relative ease in the ability to produce genetically modified animals [11, 12]. In order to study the molecular pathways and the progression of small animal AAA, a wide variety of imaging modalities have been employed. These include ultrasound, computed tomography (CT), magnetic resonance (MR), near-infrared fluorescence (NIRF), bioluminescence, single-photon emission computed tomography (SPECT), and positron emission tomography (PET). Each modality has strengths and weaknesses, making recently developed combined imaging systems (such as SPECT/CT, PET/CT, and PET/MR) attractive alternatives as they become readily available.

Significant challenges exist in adapting clinical imaging systems for small animal use. Considerations can include radioactive dose requirements, body mass, anesthesia procedures, and contrast infusion techniques, all of which can differ greatly from a clinical setting [13]. New challenges arise while engineering dedicated small animal systems. For example, PET image resolution must be significantly higher with a small animal scanner than with a clinical system [14]. With small animal ultrasound, the signal-to-noise ratio and tissue contrast are often insufficient when imaging mice and rats [13]. In spite of these challenges, some imaging systems scale favorably for small animals. For example, the static MR field strength can be higher and the receiving coil can be closer with small bore scanners, both of which lead to an increased signal-to-noise ratio. In addition, *in vivo* optical imaging is easier in small animals due to the decreased path-length photons are required to travel. Small animal imaging has become an important tool in preclinical aneurysm research.

In this review, we highlight the recent evolutions in small animal AAA models induced via exogenous chemicals and genetic disruptions. We also describe established anatomical and molecular imaging methods, address clinical translation, and identify possible future approaches to small animal AAA imaging. The work highlighted in this review is mostly intended to characterize aneurysm progression through the use of small animal imaging, with the hope of one day leading to improved clinical AAA treatment.

## 2. Small Animal Models

*Exogenous Chemical Induction.* The three most common mouse models for exogenous chemical induction of AAA use pancreatic porcine elastase, calcium chloride (CaCl_2_), or angiotensin II (AngII).

### 2.1. Elastase

Elastase-induced AAA in animal models was developed from early clinical data suggesting that elastin degradation played a significant role in AAA formation [15, 16]. Clinical pathology showed elastin structure deficiencies and high elastase activity in aneurysmal tissue. This led to early use of luminal perfusion with porcine pancreatic elastase within rats to induce aneurysms [17]. Higher concentrations of elastase led to more severe elastic tissue damage and arterial dilation. AAAs have been produced within the murine infrarenal aorta by utilizing porcine pancreatic elastase administered via an inserted catheter at the iliac bifurcation [18]. Elastase leads to elastin fiber degradation and higher levels of MMP-2 and MMP-9 expression. Though this is a transluminal perfusion, the outer adventitia is thought to play a role in the inflammatory signaling macrophage cascade that follows [9]. Other work with mice deficient for MMP-12 [18] or IL-17 [19] showed reduced aortic dilation. Interestingly, gender research revealed that male rats formed larger AAAs at a higher rate of incidence, while females were partially protected through estrogen-mediated reductions of MMP-9 production [20]. Successful elastase variations have led to further characterization of this murine AAA model. For example, porcine pancreatic elastase applied periadventitially also produces an AAA and is an easier surgical procedure [21].

### 2.2. Calcium Chloride

To induce a murine CaCl_2_ aneurysm, a small gauze saturated in a CaCl_2_ solution is placed centrally onto the exposed abdominal aorta, resulting in intimal and medial macrophage infiltration and significant diameter increase 14 days after surgery [22]. Researchers hypothesized that calcium chloride treatments disrupted the targeted elastin network from calcium precipitates, thus activating inflammatory pathways also observed in the human AAAs.

Further genetic characterization focused on the effects of MMP activity within transgenically modified mice. Mice deficient in MMP-9 (macrophage cell derived) or MMP-2 (mesenchymal cell derived) were treated with CaCl_2_ in order to induce AAAs [23]. After 10 weeks, no aneurysm formation was seen in either knockout model. Interestingly, the competent macrophages from wild-type controls were then infused into the corresponding knockout, resulting in AAA reconstitution in MMP-9^−/−^ animals, but not MMP-2^−/−^ mice [23].

### 2.3. Angiotensin II

The AngII model creates suprarenal AAAs by continuously infusing AngII subcutaneously in low density lipoprotein receptor (ldlr^−/−^) or apolipoprotein E (apoE^−/−^) knockout mice [24]. This vessel expansion was unexpected and the mechanisms that lead to the dilation are still not fully understood. AngII doses did not alter arterial blood pressure, murine body weight, serum cholesterol concentrations, or lipoprotein-cholesterol distribution [25]. The addition of doxycycline, a broad-system MMP inhibitor, reduced AAA incidence by 51% within AngII-induced murine aneurysms [26, 27], even though no effects were observed on blood pressure or serum cholesterol concentrations. Other studies have tried to define the role that AngII-induced hypertension plays within this AAA model. For example, ldlr^−/−^ and apoE^−/−^ mice were infused with AngII or norepinephrine, producing similarly increased blood pressures for both groups. Aneurysm formation and atherosclerotic lesion incidence in AngII-infused mice increased, while norepinephrine-infused mice did not show an increase in vessel diameter or atherosclerotic lesions [28]. Administration of hydralazine (a vasodialator) reduced systolic blood pressure without slowing AAA formation or reducing atherosclerotic lesion incidence.

Much is already known about the pathophysiology underlying the AngII/apoE^−/−^ AAA model. Infrarenal medial macrophage accumulation and medial dissection are the first stage of AngII-induced murine AAA progression, acting as a stimulus for elastin degradation [29]. Vascular hematoma, macrophage infiltration [25], and thrombus formation, all of which are common in human AAAs, typically occur after 3–10 days in the murine model. Remodeling and aneurysmal tissue generation follow, associated with elastic fiber regeneration, luminal surface reendothelialization, and aneurysmal neovascularization. Recently, transforming growth factor beta (TGF-*β*) was shown to limit the innate immune response and preserve murine vessel integrity, as inhibition of this growth factor enhanced monocyte invasiveness and MMP-12 activity [30]. This work has identified TGF-*β* as a potential therapeutic for the treatment of aneurysms and should be further investigated.

### 2.4. Gene Disruption

AAAs within small animal models have also been induced through the use of engineered genetic manipulations. Extracellular matrix maturation defects disabling collagen and elastin crosslinking have been shown to increase AAA susceptibility [31]. Genetic inactivation of lysyl oxidase (Lox), an extracellular copper enzyme initiating collagen and elastin crosslinking, resulted in aortic aneurysm development through fragmented elastic fibers and high rupture rates [32]. However, these animal models often develop thoracic aortic aneurysms (rather than AAAs), limiting their usefulness for abdominal investigations [9].

Genetic inhibition of MMPs is a common mechanism to study AAA progression within all three aforementioned chemically induced murine models. TIMP-1 deficient mice produced pseudomicroaneurysms and medial rupture, stemming from macrophage infiltration of medial elastic lamellae [33, 34]. Some recent genetic MMP characterization has focused on the overriding effect of membrane type-1 MMP (MT1-MMP) with the CaCl_2_ model. Investigators utilized MT1-MMP^−/−^, MMP-2^−/−^, and MMP-9^−/−^ knockout mice to investigate extracellular matrix breakdown [35]. They found lymphocyte infiltration was greatly reduced in the MT1-MMP^−/−^ model, indicating that MT1-MMP plays an unexpected but dominant role in macrophage-mediated elastolysis. Other work has shown that MMP-2 and MMP-9 expression can be attenuated due to tumor necrosis factor-alpha (TNF-*α*) deficiency, indicating that TNF-*α* plays a central role in regulating matrix remodeling during AAA formation [36].

Evidence that inflammation plays an important role in aneurysm progression has been confirmed time and again. Mast cells, a key component of the inflammatory process, are thought to contribute to AAA pathogeneses in mice, primarily through release of the proinflammatory cytokines interleukin-6 (IL-6) and interferon-*γ* (IFN-*γ*). Investigations done using IL-6 and IFN-*γ* knockout mice showed that the inflammatory cascade produced lesions in the smooth muscle cell layer, leading to aortic smooth muscle cell apoptosis, matrix-degrading protease expression, and vascular wall remodeling [37]. Disruption of IL-1*β* signaling, via a genetic deletion, significantly protects against disease progression in the elastase-perfusion model [2]. Elastin preservation and reduced macrophage and neutrophil infiltration suggest that IL-1*β* disruption could be used as a novel AAA therapeutic strategy. Similarly, platelet receptor inhibition within the AngII murine model limited AAA progression, macrophage infiltration, and MMP production [38]. Thus, there is a growing amount of evidence in a variety of animal models that supports the key role inflammation plays in AAA disease.

## 3. Anatomical Imaging

Monitoring the progression or regression of aneurysms has become easier due to recent developments in vascular imaging methods. The most frequent clinically utilized technique is ultrasound imaging. Other conventional imaging modalities that produce high-resolution images are computed tomography (CT) and magnetic resonance (MR) imaging. Researchers should choose the imaging technique that is best for their work based on the modality's strengths and weaknesses. For small animal AAA research, the use of multiple imaging modalities can often provide more information that can be used to characterize mechanistic and physiological progression.

### 3.1. Ultrasound

Ultrasound is the standard technique for diagnosing and monitoring nonruptured AAAs in the clinic [39]. It is noninvasive, accurate, reproducible, fast, uses no ionizing radiation, and is widely available to clinicians, making it possible to continuously monitor AAA progression and development over time [10]. It involves a transducer placed against skin that emits high-frequency sound waves, which are then reflected back by internal organs to produce ultrasound images. The effective contrast depends on a number of factors such as sound speed, sound attenuation, back scatter, and imaging algorithms [40]. Ultrasound is close to 100% sensitive for detecting aneurysms with a diameter greater than 30 mm [41, 42] and also provides information on size and shape of intraluminal thrombi [43].

**Figure 1:**
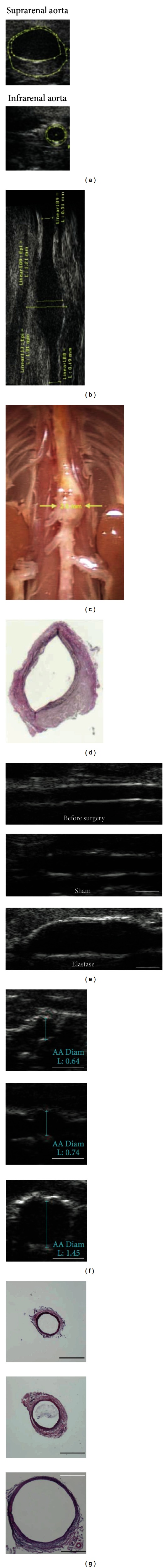
Example of high-frequency anatomical ultrasound images of abdominal aortas obtained noninvasively. ((a)–(d)) Images of a suprarenal angiotensin II-induced abdominal aortic aneurysm (AAA). (a) Transverse ultrasound images of suprarenal and corresponding infrarenal aorta. (b) Longitudinal view of a suprarenal AAA. (c) Dissected abdominal aorta for anatomical comparison and (d) histological cross-section of the suprarenal aorta from the same animal in the dilated region. ((e)–(g)) Images of elastase-induced AAAs. (e) Longitudinal images of the vessel before surgery, after sham surgery, and after intraluminal elastase perfusion. (f) Corresponding transverse images and (g) histological cross-sections stained with H & E. Figure adapted from [45] for ((a)–(d)) and [46] for ((e)–(g)).

Wang et al. (2001) were the first to use ultrasound technology to measure aneurysms noninvasively in mice [44]. Since then, ultrasound has witnessed tremendous progress. More recently developed commercially available high-frequency ultrasound imaging systems (VisualSonics Inc., Toronto, Canada) can provide increased spatial resolution and make it possible to apply ultrasound for the accurate quantification of aortic diameter and wall thickness in mice [45–49]. Others have measured aortic diameter *in vivo* using transabdominal 40 MHz B-mode imaging of AngII-induced AAAs [48]. High-frequency ultrasound was also successfully used to show that suprarenal aortic expansion occurs rapidly after initiation of AngII infusion [50]. Examples of transverse and longitudinal ultrasound images showing a murine AngII-induced AAA are shown in Figures 1(a) and 1(b). These measurements made with ultrasound were confirmed by a postmortem examination and histological sectioning of the abdominal aorta, as reported by Martin-Mcnulty et al. [45]. Another example of longitudinal and transverse ultrasound imaging using the elastase model is shown in Figures 1(e)–1(g), as reported by Azuma et al. They assessed the utility of high-frequency ultrasound measurements of aortic lumen diameter, eliminating the need for sacrifice required for *in situ* microscopy [46].

The ability of ultrasound to diagnose and characterize AAAs has been improved recently through the development of several advanced imaging techniques: speckle tracking, three-dimensional ultrasound imaging, Doppler imaging, and pulse wave velocity measurements. Speckle tracking has been used to quantify asymmetry and circumferential strain in AngII-induced AAAs [51]. Three-dimensional ultrasound imaging systems are useful when measuring aneurysm length, diameter, and volume [52, 53]. Dynamic properties of vessels can also be measured by ultrasound using M-mode or other tracking features [54], thus obtaining additional information about the distensibility of aneurysms [55]. Tissue Doppler imaging is an ultrasound technique that can measure *in vivo* wall motion along an arterial segment [45, 56–59]. Since traditional ultrasound sensitivity is limited, improvements have been reported through the use of color duplex ultrasound scanning and contrast-enhanced ultrasound [60, 61]. Finally, a more recent technique using pulse wave velocity (PWV) can accurately indicate changes in AAA wall properties (and possibly AAA rupture potential) by measuring the velocity of pressure waves generated by the left ventricle as it travels down the aorta [62, 63]. Although useful, aortic PWV does not provide localized data, something that MR and computed tomography (CT) imaging can acquire [64].

While ultrasound still remains the most common technique for imaging AAAs, it does have its limitations. Complicated and tortuous geometries are more difficult to evaluate with ultrasound than with cross-sectional imaging techniques due to limited resolution and limited signal-to-noise ratio. Furthermore, artifacts from bowel gas and obesity can limit the use of ultrasound [10]. Thus, CT and MR (as described in the following sections) can provide certain advantages [65].

### 3.2. Computed Tomography

Computed tomography (CT) can produce high-resolution three-dimensional images of internal objects and can measure aortic diameter with more precision than ultrasound [66]. Multiple X-ray images are taken around a single axis of rotation and then reconstructed to produce an anatomical image [67]. Apart from use in preoperative diagnosis [68], CT is also the preferred clinical method for small aneurysm followup largely due to great spatial resolution, speed, and reproducibility. Radiation dosages can be a concern, but risks are minimized if imaging is managed appropriately [10, 69]. Contrast-enhanced spiral CT angiography is often used for preoperative planning and evaluation prior to treating AAA patients with stent grafts [65, 67]. It can be used to measure the maximal transverse diameter of the aneurysm, identify major branching arteries, detect the presence of intraluminal thrombus, and determine the extent and calcification of tortuous vessels [43]. In current clinical practice, both ultrasound and CT are used as surveillance imaging tools, especially in the early followup. Generally, when the aneurysm sac begins to shrink, it is said to be reasonable to move from CT to ultrasound due to radiation dosage concerns, thus reserving CT for high-risk patients [69].

Reliable and accurate 3D geometrical models of the murine aorta have been reconstructed using *in vivo* micro-CT with a vascular contrast agent (Fenestra VC-131) [70]. Trachet et al. went further and developed an experimental-computational framework combining information from both contrast-enhanced micro-CT (arterial geometry) and high-frequency ultrasound (for flow boundary conditions) to set up mouse-specific computational fluid dynamic simulations in apoE^−/−^ mice [71, 72]. This work provides insight into the differences in flow characteristics between mice and humans that may help elucidate the suprarenal location of AngII-induced aneurysms.

### 3.3. Magnetic Resonance Imaging: Anatomical

Magnetic resonance (MR) imaging allows accurate detection of AAA without requiring contrast agents, intravascular catheter, or ionizing radiation. Similar to CT angiography, MR provides high-resolution 3D anatomical imaging of aneurysms in both humans and small animals [73]. MR takes advantage of the fact that atoms with angular momentum, such as the hydrogen atoms in water, can be thought of as charged spheres with a small magnetic moment. The combination of a static magnetic field with overlaid magnetic field gradients can produce an image by identifying the signal's origin. In biological tissue, proton-proton and proton-tissue interactions can produce useful contrast [74]. Differing tissue relaxation times gives rise to endogenous MR contrast, where T1- and T2-weighted spin echo sequences can be used to clearly identify the various constitutive AAA layers.

**Figure 2:**
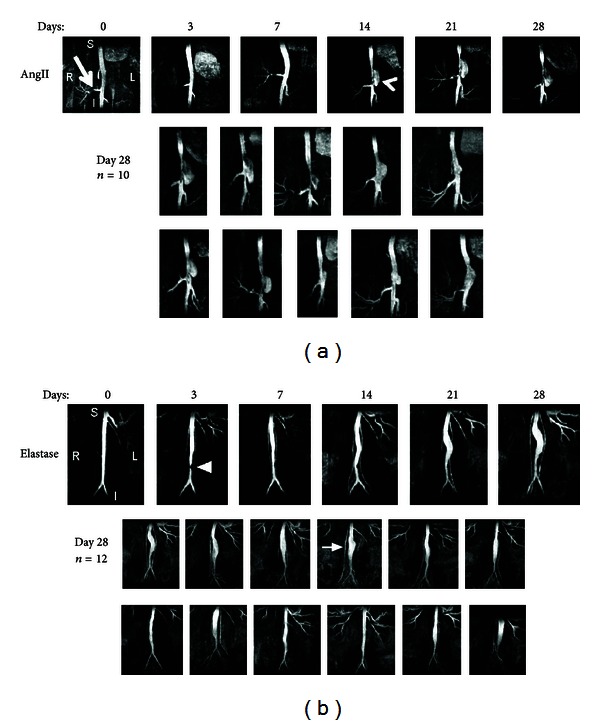
Coronal magnetic resonance maximum intensity projections showing lumen expansion in (a) angiotensin II-induced (AngII) and (b) elastase-induced abdominal aortic aneurysms (AAA). Angiotensin II-induced AAAs appear suddenly (arrowhead) and expand leftward directly above the right renal artery (arrow). Ten additional angiotensin II AAAs are shown at day 28. Elastase-induced AAAs expand slowly. Small region of signal hypointensity is seen at day 3 (triangle) due to a suture in the vessel. Twelve additional elastase AAAs are shown at day 28. The testicular artery is highlighted (arrow). Figure adapted from [77].

MR has emerged as a leading noninvasive *in vivo* imaging modality to assess AAA morphometry in mice *in vivo* [64, 75–77]. For example, MR was used to assess vessel dilation for both AngII and elastase-induced aneurysms in mice over a four-week period, as highlighted in Figure 2. Temporally and spatially resolved data quantifying murine aortic motion and curvature *in vivo* was obtained by Goergen et al. in apoE^−/−^ mice by acquiring time-of-flight MR angiography [64]. Choke et al. successfully used MR along with microscopy to determine maximum diameter and cross-sectional area of AAA in apoE^−/−^ mouse models [78]. Klink et al. also demonstrated use of high-resolution, multisequence MR to characterize the temporal progression of an AAA in AngII infused mice [79]. In addition, *in vivo* phase-contrast magnetic resonance (PCMR) velocity measurements were used by Amirbekian et al. to characterize the hemodynamic environment of the suprarenal and infrarenal abdominal aorta of normal and apoE^−/−^ mice [80]. Longitudinal high-resolution MR scans have recently been used to characterize aneurysm development in murine elastase-induced AAAs without significant mortality, measuring inner lumen diameter, outer vessel diameter, and vessel wall thickness [73].

Modified MR techniques can be applied to provide more information than just vessel expansion. In patients, dynamic gadolinium-enhancement techniques have been shown to help characterize AAA [81]. Magnetic resonance angiography has also been used in the clinic to characterize aortic stiffness and elastic modulus as indices of arterial wall compliance during the cardiac cycle [82]. Furthermore, the assessment of aortic flow patterns has been made possible by the development of 3D, time-resolved, phase-contrast, velocity-mapping magnetic resonance sequences which allows for acquisition of shear-stress measurements of the arterial wall [83]. However, the clinical relevance of these techniques continues to be explored [43].

It is now evident that a variety of anatomical imaging modalities are capable of tracking AAA progression in both humans and small animals. Yet other less developed molecular imaging techniques may provide more information that could be used to better predict AAA progression, an area of certain clinical interest. Indeed, the development of radiotracers that target specific molecules involved in AAA growth or the use of molecular optical imaging could provide new insights into aneurysmal disease.

## 4. Molecular and Functional Imaging

### 4.1. Magnetic Resonance Imaging: Molecular

Molecular MR can explore events that occur at cellular and subcellular levels with nanomolar sensitivity [84]. Anatomical MR, even when using contrast agents, has only micromolar sensitivity [85]. Thus, the development of new molecular contrast agents is an area of current research aiming to increase the capabilities of magnetic resonance [86].

One of the more established uses for molecular MR in AAA research is monitoring macrophage accumulation. Ultrasmall superparamagnetic iron oxide (USPIO) particles are a useful label for macrophages with phagocytic activity, although surrounding tissue uptake of USPIOs can also cause some imaging difficulty [87]. In general, iron oxide particles reduce the T2 relaxation time of nearby absorbing tissues [88, 89]. To increase *in vivo* macrophage imaging sensitivity, iron oxides (specifically heavy chain ferritin or HFn) were attached to Arg-Gly-Asp (RGD), a short integrin binding sequence that has been used to image atherosclerosis [90]. HFn molecular imaging is enhanced with RGD targeting, extending imaging capabilities to atherosclerotic macrophages and angiogenic endothelial cells [90]. Similar studies have been done clinically with similar results, as human AAAs also show uptake of USPIOs which is suggestive of inflammation [91].

Contrast agents specific to MMPs have been developed and used to further explore the molecular processes associated with AAAs. P947, a recently developed MR contrast agent, was created to target atherosclerotic plaque by coupling an MMP inhibitor to a gadolinium chelate (Gd-DOTA) [92]. Gd-DOTA and other gadolinium chelates enhance MR imaging by shortening the T1 relaxation time of nearby protons [93]. P947 was shown to have higher affinity for MMPs than Gd-DOTA alone, particularly within more stable plaques [92]. P947 greatly enhanced the MR signal in atherosclerotic vessel walls of the apoE^−/−^ mice, significantly more than either its untargeted counterpart (a scrambled form of P947) or a Gd-DOTA control [94]. In an elastase-induced AAA model, P947 has shown enhanced MMP targeting in AAA MR imaging when compared to either control [95]. In all of the P947 studies, areas with P947 enhanced MR image contrast also had a variety of active MMPs.

Another contrast agent, the collagen-specific protein CNA-35, has been used in the AngII-induced AAA model. Micelles of CNA-35 were created as the contrast agent, while a mutant version was used for comparison [79]. Addition of CNA-35 micelles enhanced MR signal in the aneurysm wall, associating with the breakdown of collagen during aneurysm progression. This property can also be used to differentiate between collagen rich and collagen poor AAAs [79].

**Figure 3:**
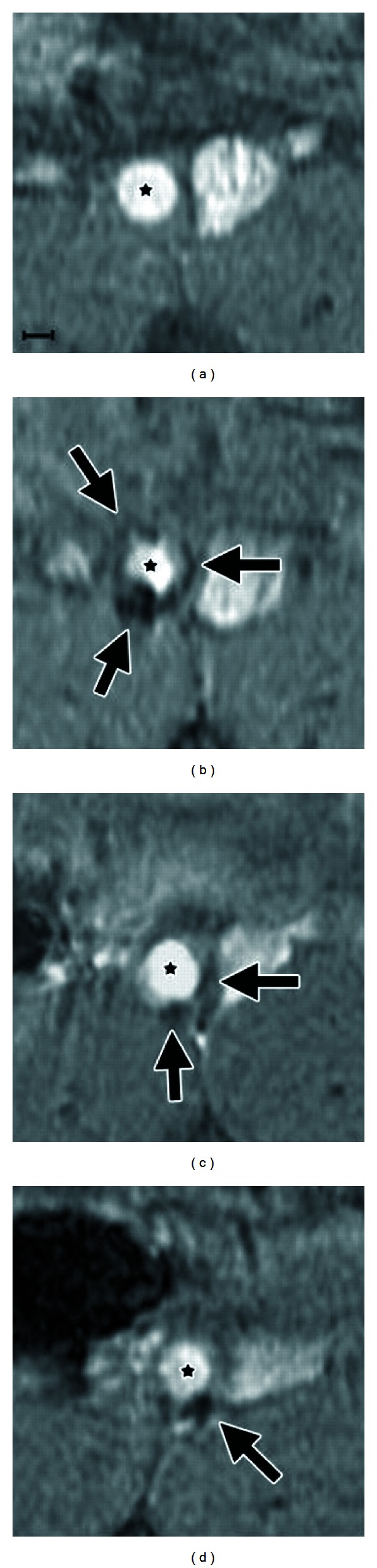
Transverse T2*-weighted magnetic resonance images, the spin-spin relaxation time measured in gradient echo sequences, of a single murine abdominal aortic aneurysm prior to iron oxide nanoparticle-labeled vascular smooth muscle cell delivery (a) and on postdelivery days 0 (b), 21 (c), and 28 (d). Arrows represent areas of hypointense signal in the aortic wall. The vessel lumen is highlighted with (★). Scale bar represents 1 mm. Figure adapted from [96].

MR can also be used to track cellular implantation and migration. One such example is the uptake of iron oxide nanoparticles into vascular smooth muscle cells (VSMCs) [96]. One recent study examines how iron oxide nanoparticles (IONPs) change the therapeutic effects of VSMCs [96]. Not only do IONP-VSMCs show the same efficiency of cell delivery as VSMCs alone, but they can also be detected with MR imaging. This allows for unhindered monitoring of VSMCs that have migrated in or near the AAA. As shown in Figure 3, the use of IONP-labeled VSMC is effective in revealing the labeled smooth muscle cells collecting around an aneurysm [96]. All parts of Figure 3 are taken from the same animal, verifying that IONPs can be used as an effective *in vivo* contrast agent.

While every imaging modality has its own advantages, combining various approaches allows for more imaging capabilities. For example, characterizing macrophage accumulation in AAAs can be done using molecular MR complemented with bioluminescence. Super paramagnetic iron oxide (SPIO) nanoparticles and transgenically modified luciferase expressing macrophages can be used to quantify inflammation with MR and bioluminescence [97]. Both modalities confirmed macrophage accumulation at the AAA and also showed that a majority of macrophages ended up in the adventitia.

One of the main advantages of MR is its noninvasive nature. The ability to acquire images at multiple timepoints within the same animal reduces the overall number of animals needed and provides evidence for disease progression over time. Molecular MR is currently limited in measurement capabilities. Contrast agents are available to target a handful of molecules and cells, but there are many applications yet to be explored. Developing new and innovative contrast agents and dual modality imaging strategies are both areas that could lead to further imaging advancements.

### 4.2. Near-Infrared Fluorescence

There are three general parameters that describe the interaction of photons with biological tissues: absorption of light, scattering of light, and fluorescence emission. The light absorption by endogenous chromophores in living tissues (including hemoglobin, melanin, and lipid [98–101]) is typically within the visible spectral region (400–700 nm), limiting penetration to only a few millimeters. Additionally, light absorption due to water is notably increased above 900 nm [98]. Photons in the near-infrared (NIR) range (700–900 nm), however, penetrate deeper than visible light [99]. Hence the NIR window is often utilized in biomedical imaging, as photons have a penetration depth of several centimeters in tissue [98–101]. For example, near-infrared fluorescence (NIRF) and bioluminescence imaging are both highly sensitive techniques that can provide molecular information [102–106].

Indocyanine green, the only currently FDA-approved NIR cyanine fluorescent dye, has been used to assess microvasculature in tumors [107, 108], lymph nodes [109, 110], and atherosclerotic plaques [111], but has not yet been applied to AAAs. More recent work has used indocyanine green to image cerebral aneurysms during surgery [112, 113], providing hope that fluorescent imaging of abdominal aneurysms may also be useful. The location of the aorta certainly provides challenges, but access through catheters or during open surgical procedures could certainly help overcome some of the technical challenges associated with optical imaging in the clinic.

**Figure 4:**
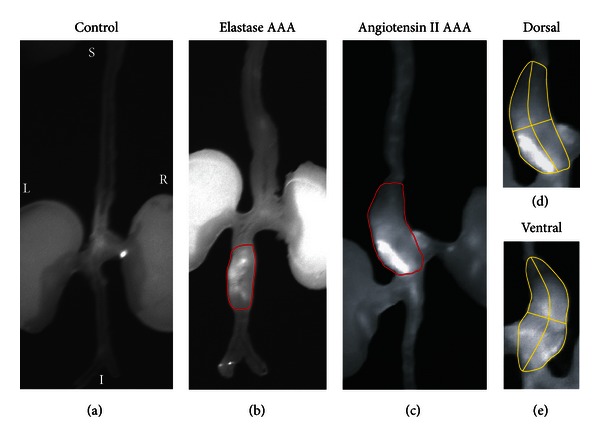
Near-infrared fluorescence (NIRF) image of both angiotensin II-induced and elastase-induced aneurysms. (a) Control apolipoprotein-E deficient mouse aorta after injection of MMPSense 680. (b) Infrarenal aortic aneurysm induced via elastase infusion. (c) Suprarenal aortic aneurysm induced via angiotensin II infusion. (d) Dorsal and (e) ventral NIRF images of angiotensin II-induced abdominal aortic aneurysms showing asymmetric probe accumulation, suggestive of regional differences in protease activation and inflammation. Figure adapted from [77].

In preclinical research, some of the most common activatable optical probes are those sensitive to protease activation. By using the MMP-activatable probe MMPSense (PerkinElmer, Waltham, MA, USA), a direct, linear relationship between proteolytic activity and aneurysmal growth was shown through *in vivo* imaging with the CaCl_2_ model [114]. Moreover, Kaijzel et al. showed increased MMP activity in fibulin-4 mice, well before the aneurysm had actually formed [115].  Figure 4 highlights the spatial distribution of relative MMP activity showing a correlation between inflammation and fluorescent signal, a result that was also seen with a cathepsin sensitive probe [77]. These data suggest that protease accumulation and activation are increased in regions of vessel remodeling.

Transmural inflammation and adventitial neovascularization are pathological characteristics of AAAs. Previous work using *in situ* NIRF imaging revealed significant vascular endothelial growth factor receptor (VEGFR) expression in the AngII model [48]. These results showed that mural VEGFR expression, as measured with fluorescence imaging and immunohistochemistry, increased in a diameter-dependent fashion with AAA progression. This study then went further and showed that an angiogenesis inhibitor decreased the inflammatory response and attenuated AAA formation [48], results that suggest that angiogenesis inhibition should be further explored as it may expand therapeutic alternatives for the treatment of AAA disease.

Inflammation is seen in the vast majority of AAAs, making vascular inflammation-targeted imaging an active area of research. As mentioned previously, Kitagawa et al. evaluated the inflammation process in the AngII model using RGD-conjugated HFn that were also labeled with fluorescent Cy5.5 [90]. AAAs showed higher fluorescent signal intensity when compared to surrounding tissue, and these results were confirmed with immunohistochemistry [90]. These data suggest that targeted human ferritin might be a useful platform for vascular inflammation imaging in humans.

### 4.3. Bioluminescence

**Figure 5:**
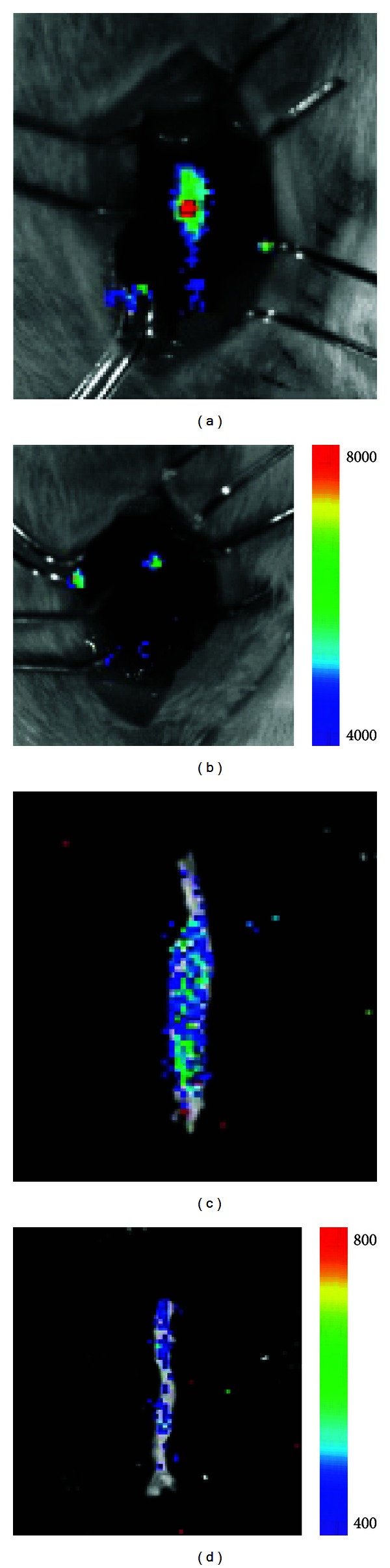
((a), (b)) *In situ* and ((c), (d)) *ex vivo* bioluminescent images of an infrarenal abdominal aortic aneurysm ((a), (c)) and control vessel ((b), (d)), with arbitrary units. Aneurysms were induced via elastase perfusion. Figure adapted from [97].

Bioluminescence imaging uses cells that have been transgenically engineered to express luciferase. These cells are implanted in animals, allowed to proliferate and produce light when they come in contact with luciferin [106].  Figure 5 shows the application of bioluminescence to study macrophage accumulation in the elastase-induced aneurysm model [97]. Macrophages from transgenic mice expressing luciferase were injected in mice from both diseased and control groups. Both the *in situ* and *ex vivo* bioluminescence images show high macrophage accumulation in AAAs when compared to control mice (Figure 5). While bioluminescence excels at providing information about the location of transgenically modified cells, image resolution and sensitivity degrade as tissue depth increases [116]. Complications associated with implantation of genetically altered cells into humans also limit the use of bioluminescence for noninvasive characterization of AAAs in the clinic.

### 4.4. Radionuclide Imaging

For the purposes of molecular and functional imaging of AAA, single-photon emission computed tomography (SPECT) provides many advantages and has become a powerful tool. Unlike contrast agents that alter image contrast, radionuclide imaging uses radioactive tracers as imaging agents. The objective of SPECT is to collect *γ* and X-ray signals from radiopharmaceuticals within the body and use these signals to create 3D tomographic images [117]. These images represent the biodistribution of the radionuclides and are obtained noninvasively. In SPECT imaging, gamma photons are emitted and lead collimators, designed to reject photons, measure the signal that is produced within a small range of angular incidence. This measurement method results in low geometric efficiencies (~0.01%) compared to positron emission tomography (PET), which uses a coincidence-detection method and eliminates the need for collimators. Due to the nature of positron annihilation, PET systems measure two photons emitted in opposite directions. For PET, the geometric efficiency is on the order of ~1% [118]. However, SPECT systems use less expensive and more stable radionuclides and can complete scans faster than PET systems [119]. Similar to SPECT, PET uses radionuclides to construct 3D images. The main advantage of PET over SPECT systems includes improved resolution, though rapidly decaying radionuclides create logistical difficulties [119].

### 4.5. Single-Photon Emission Computed Tomography

A variety of radionuclide tracers have been used to obtain molecular images of AAA using SPECT. In the late 1970s, red blood cells labeled with technetium-99m (^99m^Tc) were used to image AAA in human patients [120]. Also, ^99m^Tc-fucoidan was used to detect aneurysmal intraluminal mural thrombi. This is due to the high affinity that fucoidan has for P-selectin, an adhesion molecule whose expression is involved in aneurysmal pathophysiology [121]. Luminal thrombi in murine AAAs were also imaged using ^99m^Tc-annexin. ^99m^Tc-annexin works by binding to phosphatidylserine on apoptotic cells and activated platelets [122]. ^99m^Tc-labeled antismooth muscle myosin antibodies have shown promise for the imaging of dissecting aneurysms, as previous research showed that this tracer localizes to aortic dissections in a rat model [123]. Platelets labeled with indium 111 (^111^In) have shown small animal aortic imaging possibilities as well [124]. However, in human patients, ^111^In has exhibited a propensity to sequester in the spleen, liver, and bone marrow, causing reduced aneurysm accumulation [125]. Thallium 201 (^201^Tl) is commonly used as a myocardial perfusion agent [126]. Myocardial perfusion in patients with AAA has been quantitatively evaluated using ^201^Tl, suggesting that this tracer may be useful in predicting AAA rupture. SPECT can be used to detect multiple radionuclides at once, making it possible to analyze multiple molecular processes and giving it a distinct advantage over other modalities [127].

In order to give anatomical context and to improve quantitative SPECT data, SPECT systems are often coupled with CT [128]. This coupling has proven very useful due to the fact that functional images of the thorax and abdomen provide few landmarks to correlate with the surrounding anatomy. SPECT imaging has been used for molecular, functional, and anatomical imaging in a wide variety of areas including myocardial perfusion, cancer diagnosis, functional brain imaging, bone imaging, Alzheimer's diagnosis, and quantifying thrombus formation in AAAs [122, 129–134]. Over the past ten to fifteen years, dedicated small animal SPECT systems have been developed to aid in preclinical research [135]. This advancement has made it possible for researchers to observe molecular processes in a single animal over time. Traditionally, snapshot molecular data was collected via tissue sectioning, microscopy, or *γ* counting after euthanasia. As small animal models for AAA continue to develop and improve, SPECT systems have become increasingly valuable in research.

Despite the promise that SPECT presents for AAA molecular and functional imaging, it has several limitations, including low geometric efficiency of collimators, subpar attenuation compared to PET, low temporal resolution, inability to eliminate crosstalk between radionuclide tracers, and inefficient reconstruction software [118]. While some solutions to these issues have been addressed, the complexity and cost of SPECT and SPECT/CT systems slow improvements. Recent SPECT advances include improved scintillators, solid-state photon transducers, attenuation correction, improved image reconstruction, and dynamic SPECT [117]. As these improvements are implemented, SPECT will continue to be an attractive modality for molecular and functional imaging of AAA.

### 4.6. Positron Emission Tomography

**Figure 6:**
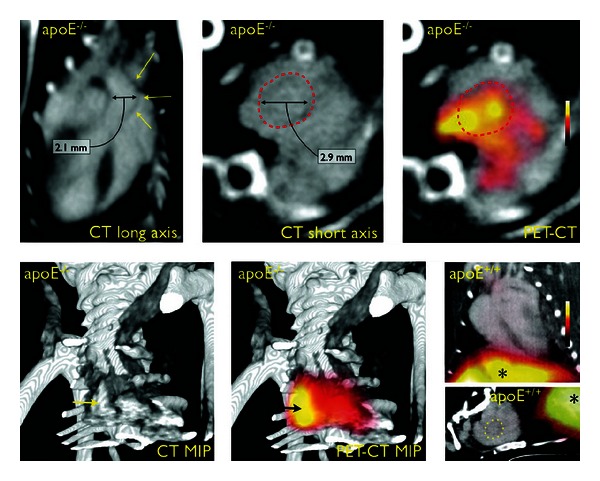
PET/CT imaging in mice with aortic aneurysms induced via angiotensin II infusion. Apolipoprotein E-deficient (apoE^−/−^) mice were compared to wild-type controls (apoE^+/+^). Dotted lines and yellow arrows outline the aneurysmal aorta. Liver signal highlighted with (∗). PET/CT images illustrate the ability of CT to add anatomical context for PET images. Figure adapted from [137].

As with SPECT, the addition of CT to stand-alone PET systems helps provide anatomical context to the generated images. Most PET/CT systems consist of both systems working in tandem and scanning sequentially, although some are able to scan simultaneously [136].  Figure 6 shows a series of images obtained from a PET/CT system [137]. In that study, fluorine-18 (^18^F) labeled nanoparticles were used to target macrophages that are a marker of inflammation in the murine AngII/apoE^−/−^ model. These results demonstrate the ability of CT to give anatomical context to otherwise ambiguous PET images.

In a similar study, ^18^F-labeled fluorodeoxyglucose (FDG), ^18^F-fluoromethylcholine (FCH), and ^18^F-DPA714 (a peripheral benzodiazepine receptor antagonist) were used to target inflammation and image AAAs in rats [134]. Results indicated that sensitivity was higher for FDG-PET than FCH and DPA714 PET, increasing interest in the FDG tracer for aneurysm imaging. High uptake of FDG is attributed to inflammation in the aneurysm wall and is thus a common tracer for molecular imaging of AAA [138, 139]. Researchers have also tried to correlate FDG uptake with aneurysm rupture risk in humans; however, quantifying this relationship has proven to be nontrivial [140]. In another study, FDG was used with PET/CT to investigate aneurysm wall pathology and coincidentally identified concomitant tumors in several patients [141]. This discovery illustrates the diversity of applications for PET/CT.

The future of PET imaging is largely in the development of new molecular probes and in improved multimodality systems (PET/CT and PET/MR). Development of new tracers is a very complex process in which the desired target must be matched with the proper targeting ligand and optimized for high efficiency binding. Due to the short lifespan of radioisotopes, proficient labeling of new tracers to peptides and antibodies requires specialized personnel, often unavailable in small medical research facilities. In addition, a suitable radionuclide must be selected based on availability, half-life, binding capability, and specific activity [142]. This process can be lengthy, arduous, and expensive. Small animal PET can act as a means to test new tracers for efficacy in a preclinical setting. Multimodality PET systems such as PET/CT (and more recently PET/MR) seem to be the future of PET molecular imaging. Researchers and clinicians alike immediately embraced PET/CT, but limitations remain and improvements continue to be developed in the areas of device hardware, reconstruction software, and detector sensitivity [143]. Progress in the area of radiopharmaceuticals is slow as regulations for clinical trials are stringent for radioactive materials. Radiotracers that have already been developed must endure extensive approval processes before human testing can occur. As these advancements are implemented, molecular and functional imaging for AAA will significantly improve.

## 5. Conclusions and Future Possibilities

Several imaging modalities applied to small animal AAA have been reviewed in this paper (see Table 1). Well-established imaging methods, such as ultrasound, CT, and MR, can provide anatomical images of mouse and rat AAAs. They have been extensively applied in both preclinical research and in the clinic for diagnosis and monitoring aneurysms. The use of molecular imaging to investigate AAA progression has recently become more popular, although clinical translation of these technologies remains difficult. Molecular aneurysm imaging is likely to grow as more potential AAA biomarkers are identified. The future of AAA imaging lies in several major directions: understanding underlying progression mechanisms, multimodality imaging, application or development of novel AAA imaging technology, and clinical translation of molecular imaging methods. These future directions include several aspects which should be addressed.

First, development of clinically relevant animal models is likely required to elucidate mechanisms of AAA progression. Though mice and rats are the most frequently used species for aneurysm models, many studies only observe vessel dilation through the first four weeks after AAA onset. The current technique of aneurysm screening in humans rarely identifies vascular dilation this early in AAA development, and treatment is usually started well outside of this four-week range. Additionally, there is currently no single murine model able to accurately mimic all physiological features observed in the human condition [12]. Murine AAAs rarely continue to expand until rupture, the major clinical concern in patients with aneurysms. Thus development of more realistic animal models may provide a better understanding of the underlying disease. Future small animal model refinements will also likely help guide the design and evaluation of new monoclonal antibodies or small molecules that could prevent AAA growth.

Second, novel imaging methods and contrast agents could be used to provide new insights into vascular disease. Photoacoustic (PA) imaging, which detects the acoustic wave rising from photon absorption, is one promising technology for AAA imaging. Biomedical PA imaging has superior tissue penetration due to acoustic detection and molecular specificity because it utilizes optical excitation [144, 145]. Recent studies of atherosclerosis using microscopic [146, 147] and intravascular [148, 149] PA imaging raise the intriguing possibility of using PA methods to study aneurysms. Another potential method for AAA visualization is Raman spectroscopy and imaging. The contrast from Raman spectroscopy originates from molecular vibrations, which is a direct measurement of molecular properties. Intravascular Raman spectroscopic catheters have been applied to human coronary atherosclerosis [150]. This work suggests that Raman spectroscopy could also be used to investigate the constituents of the aneurysmal wall *in vivo*.

Third, development of new contrast agents, particularly nanostructures, is also advancing the field of vascular molecular imaging. While NIRF imaging has been used to assess MMP activation, VEGF expression, and inflammation, this previous work has used fluorescent dyes that are limited by photobleaching and low quantum yields. Semiconductor quantum dots, a newly developed nanoparticle fluorescent probe, are remarkably resistant to photobleaching with much higher quantum yields when compared to standard fluorescent molecules. Although quantum dots have toxicity issues, they have presently been applied for targeted *in vivo* imaging of tumors in animal models [151, 152]. Other works utilized single-walled carbon nanotubes (SWNTs) as a murine *in vivo* contrast agent for real-time fluorescence imaging through the second near-infrared region (1000–1400 nm) [153, 154]. Due to reduced optical scattering in this window, significant resolution improvements were achieved when compared to traditional near-infrared imaging [154]. This concept is attractive to molecular imaging of AAAs, since the SWNTs can be functionalized to target specific molecules. Thus, dual molecular fluorescence imaging with quantum dots or SWNTS may provide new information when used with experimental AAA models.

Finally, intravascular imaging using catheter technology has helped many imaging techniques translate into the clinic. Intravascular imaging can define arterial wall composition with high temporal and spatial resolution and is often used to guide stent implantation in coronary arteries. Optical imaging methods are often feasible during intravascular procedures since near-infrared light can penetrate through the thickness of most arterial walls. Such optical methods, including intravascular-based NIRF [114, 155–158], NIR spectroscopy [159, 160], Raman spectroscopy [150], PA imaging [148, 149], and optical coherent tomography [157, 161], have all been shown to successfully characterize coronary atherosclerosis through selective chemical contrast. However, coronary artery and aorta sizes are quite different, making implementation of intravascular optical methods for abdominal imaging challenging. For example, blood in coronary arteries is often flushed and replaced with saline to minimize light scattering, a procedure that is likely not possible in the abdominal aorta. Engineering modifications are thus needed to optimize techniques for imaging both experimental and clinical AAAs.

In summary, this review has described ongoing efforts to characterize AAA initiation and progression through imaging a variety of small animal models. Anatomical and molecular imaging modalities continue to develop, giving researchers a better picture of AAA pathogenesis through longitudinal imaging studies. While the long-term goal of this field is to help human AAA patients, a detailed understanding of small animal aneurysm models through *in vivo* imaging should help to bring novel therapies closer to clinical application.

## Figures and Tables

**Table 1:** Summary of imaging modalities used to image small animal abdominal aortic aneurysms.

Modality	Capabilities		Application		Contrast Agents/ Radionuclide Tracers
	Anatomical	Molecular/ Functional	Benefits	Limitations	
Ultrasound	X		Rapid, accurate, low cost, reproducibility, widely available	Limited resolution, image interpretation difficult, artifacts common	Microbubbles

CT	X		Rapid, high resolution, useful for early clinical followup	Ionizing radiation, requires contrast agent	Iodine or Barium

MRI	X		Soft tissue contrast, high resolution	High cost, large equipment required	Gadolinium chelates
		X	Customizable molecular targeting, cell tracking	Limited sensitivity, requires contrast agent	USPIOs or gadolinium chelates

NIRF		X	Low cost, widely available	Photobleaching, low quantum yield, shallow tissue penetration	MMPSense, scVEGF/Cy, RGD-HFn-Cy5.5

Bioluminescence		X	High sensitivity, high specificity	Shallow tissue penetration, requires transgenic modification	Exotic transgenic cells combined with luciferin

SPECT		X	3D imaging, widely available, highly sensitive, simultaneous imaging of multiple processes	Limited temporal resolution, few radionuclide tracers	^ 99^TC, ^111^In, ^201^Tl, ^123^I, ^131^I

PET		X	Quantification of metabolism and blood flow, high sensitivity, many radionuclide tracers available	High cost, limited availability, large equipment required, short tracer half-life, single process evaluation	^ 18^F, ^11^C, ^13^N, ^15^O, ^82^RB
